# Gambling problems and help-seeking in serving United Kingdom military personnel: A qualitative study

**DOI:** 10.3389/fpsyt.2022.1003457

**Published:** 2022-12-23

**Authors:** Hannah Champion, Amy Pritchard, Glen Dighton, Simon Dymond

**Affiliations:** ^1^School of Psychology, Swansea University, Swansea, United Kingdom; ^2^Department of Mental Health and Social Work, Middlesex University, Hendon, United Kingdom; ^3^King's Centre for Military Health Research, Institute of Psychiatry, Psychology and Neuroscience, King's College London, London, United Kingdom; ^4^Department of Psychology, Reykjavík University, Reykjavík, Iceland

**Keywords:** interviews, serving personnel, gambling, alcohol, mental health, COVID-19, help-seeking

## Abstract

**Introduction:**

In military personnel are vulnerable to gambling problems, yet many are reluctant to seek help. The aim of the current study was to explore the lived experience of problem gambling and help-seeking among serving members of the United Kingdom Armed Forces.

**Methods:**

Seventeen individuals from a larger, cross-sectional survey of gambling and wellbeing in the Royal Air Force (RAF) completed semi-structured interviews. Interview questions focused on personal experiences, the context of the RAF and its influence, knowledge and experiences of treatment and support services, and the impact of COVID-19.

**Results:**

Reflexive thematic analysis revealed four themes: (1) harmful and protective occupational factors; (2) socio-cultural and personal influences; (3) organizational attitudes toward mental health and help-seeking, and (4) current support pathways and provision.

**Discussion:**

Findings also indicated that gambling and alcohol use are common within the RAF, and that personnel are actively coping with mental health challenges.

## Introduction

Serving military personnel in the United Kingdom (UK) Armed Forces, particularly those returning from operational deployment, are at heightened risk of reporting mental and physical health difficulties (1–3). For instance, the likelihood of a common mental disorder diagnosis such as anxiety or depression is approximately double in the military compared to the general population (1). Indeed, serving in the military is considered a risky, stressful occupation as it necessarily involves exposure to stressful events, extended periods away from family and friends, and an organizational culture with close social bonds and established hierarchy. In the UK, the presence of fruit/slot machines on military bases provide opportunities to gamble in addition to other, more popular forms [e.g., online gambling *via* smartphones (4)]. Overall, the nature of military service may itself represent a risk factor for risky behavior like problematic gambling and alcohol use and common mental disorders (1, 4–8).

Despite this, little is known about gambling problems in currently serving military personnel or about its associations with other risky behaviors and common mental disorders (4). A study in recently deployed Australian Defence Force personnel identified that, compared to experiencing no gambling-related problems, problematic gambling was associated with a 5-fold increase in high psychological distress, more than 6-fold increases in rates of probable depression and alcohol dependence, and a 7-fold increase in probable major depression and PTSD (9). Additionally, problem gambling was associated with a 3-fold increase in self-reported fair/poor quality of life. In a recent survey of a large sample of currently serving UK Royal Air Force (RAF) personnel, 2% indicated they had experienced problematic levels of gambling, while the risk of experiencing any gambling problems at all was associated with a young age range, male gender, and holding a non-commissioned rank (4). Moreover, those RAF personnel with severe to moderate anxiety and depression were at substantially increased risk of problem gambling. Quantitative analyses of survey data such as these chart the prevalence of gambling problems among the military. Despite this, a great deal further remains to be known about the subjective, lived experience of personnel struggling with the consequences of problem gambling and/or a co-occurring mental disorder (10, 11).

A recent scoping review of studies exploring gambling harm in active military personnel of “Five-Eyes” countries (United States, Canada, UK, Australia, and New Zealand) identified few qualitative case studies, mainly from the gray literature and policy reviews, concerning the impact of gambling in the military (12). This lack of qualitative research concerning the lived experiences of those who gamble, and the combined impact of coping with mental health difficulties while serving in the military, suggests that a robust understanding of these perspectives has yet to emerge. This gap in research knowledge may be due, at least in part, to the relationship between stigma and help-seeking for mental health difficulties within the military. Sharp et al. (13) systematic review of barriers to help-seeking in serving military personnel identified two main sentiments: that they would be treated differently by unit leadership and be seen as ‘weak.' Despite efforts to decrease stigma surrounding mental health, both in military contexts and in the general population (14, 15), considerable barriers still exist (16–18). Within the military then it is likely that stigma concerning a perceived gambling problem will exacerbate pre-existing help-seeking difficulties and act as a further barrier to treatment.

The current study therefore sought to explore the lived experience of serving members of the UK Armed Forces, some of whom were coping with gambling problems, alcohol use, and mental health help-seeking difficulties. Our aim was to gain a better understanding of how service personnel cope with the negative consequences of harmful gambling and to what extent unique organizational and cultural factors surrounding serving in the Armed Forces impacted one's ability to seek help.

## Methods

### Larger study

The present study was part of a larger, cross-sectional survey of gambling and wellbeing in the RAF (4). To participate in the larger study, participants had to be currently serving members of the RAF and at least 18 years old (*n* = 2,119). Participants completed self-report questionnaires assessing gambling experience and severity, mental health (anxiety and depression), alcohol use, substance use, and the impact of COVID-19 on their health and wellbeing.

After completing these measures, participants were invited to participate in detailed, follow-up interviews to explore their lived experience of gambling and gambling-related harms; however, mental health, alcohol use and substance use were also discussed to capture the overall wellbeing of personnel. Ethical approval was obtained from the Ministry of Defence Research Ethics Committee (Reference: 1051/MoDREC/20).

### Sample

For the follow-up interviews, 647 individuals expressed interest in participating. A total of 20 individuals were approached based on the range of their gambling severity (Problem Gambling Severity Index, PGSI) scores. The full, 9-item PGSI was used consisting of items such as “Have you ever bet more than you could afford to lose?” rated on a 4-point scale ranging from “never” to “almost always”. Scores for the 9 items are summed, and the results are interpreted as follows: 0 (non-problem gambling); 1–2 (low level of problems with few or no identified negative consequences); 3–7 (moderate level of problems leading to some negative consequences); and 8 + (problem gambling with negative consequences and a possible loss of control).

Seventeen individuals agreed to participate in an hour-long interview (three did not reply to follow-up invitations). Twelve participants identified as male, four identified as female and one identified as non-binary (Table 1). The sample comprised of two individuals experiencing problem gambling (PGSI >8), seven individuals experiencing moderate risk gambling (PGSI 3–7), one individual reporting low risk gambling (PGSI 1–2), two individuals who reported no gambling problems (PGSI 0), and five non-gamblers.

**Table 1:** Participant information.

**Participant number**	**Age category (years)**	**Gender**	**Ethnicity**	**Rank**	**Problem gambling severity index (PGSI) score category**
1	35–44	Female	White	Commissioned	Non-problem gambling
2	45–54	Male	White	Non-commissioned	Moderate risk
3	25–34	Male	White	Commissioned	Non-problem gambling
4	35–44	Male	White	Non-commissioned	Moderate risk
5	35–44	Male	White	Non-commissioned	Moderate risk
6	35–44	Male	White	Non-commissioned	Moderate risk
7	35–44	Non-binary	White	Non-commissioned	Moderate risk
8	25–34	Male	White	Commissioned	Non-problem gambling
9	35–44	Female	White	Commissioned	Non-problem gambling
10	35–44	Male	White	Commissioned	Moderate risk
11	35–44	Male	White	Non-commissioned	Non-gambling
12	35–44	Male	White	Commissioned	Non-problem gambling
13	25–34	Male	White	Non-commissioned	Problem-gambling
14	35–44	Male	White	Non-commissioned	Low-risk gambling
15	35–44	Male	White	Non-commissioned	Problem-gambling
16	25–34	Female	White	Non-commissioned	Non-gambling
17	25–34	Female	White	Non-commissioned	Moderate risk

The sample size was determined by level of interest and when data saturation [the point by which no new information or patterns are being discovered (19)] was deemed to have been reached. As this study sought the exploration of a range of issues (gambling problems, alcohol use, and mental health help-seeking difficulties across different gambling risk-levels), achieving 17 individual interviews was practical given time and staffing constraints. We hoped as many participants as possible would come forward to share their experience and overcome any stigma by being interviewed by AP. As such, we had no a priori sample size in mind and accepted that interviewing 17 RAF personnel was realistic and representative.

All interviews took place online due to COVID-19 restrictions and were conducted in March and April 2021.

### Interview guide

A semi-structured interview guide was developed to explore the experience of RAF personnel of four wellbeing topics: mental health, gambling, alcohol, and substance use (see Supplementary material). Interview questions were designed to focus on four areas of interest and how these related to the different wellbeing topics: (i) personal experience, (ii) the context of the RAF and its influence (e.g., the culture and unique features of the RAF), (iii) knowledge and experiences of treatment and support services and (iv) the impact of COVID-19. The interview guide underwent piloting with peer contacts.

### Procedures

Participants were contacted *via* email to schedule an online interview. Signed, written and vocal consent was obtained prior to the interviews, which were conducted by AP *via* Zoom or phone and audio recorded. Audio recordings were deleted once transcribed by AP; the transcription was stored on a password protected computer. Interview length averaged 1 h. Participants were assigned a pseudonym to provide anonymity and received a £20 Amazon e-voucher for their time.

### Analysis

Data was analyzed using Thematic Analysis (TA); a six-step method used for organizing, coding and theming qualitative datasets (20, 21): familiarization, coding, generating themes, reviewing themes, defining and naming themes, and writing up. We used TA as it is not bound to a specific theoretical framework and can be applied across a range of diverse studies, whilst allowing a rich exploration of shared and discrete perspectives between individuals. Thus, the flexibility of this approach was best suited for making sense of and summarizing the rich and nuanced experiences revealed by participants in this study in an organized and vigorous manner (22).

Specifically, the first step of the process began with AP and HC re-reading each transcript multiple times to ensure interview data was accurately understood. The second step involved using Microsoft Excel to manually code each segment of text across all transcripts to generate an open code-frame, which was iteratively modified throughout the transcript review (there were no pre-set codes). Subsequently, as part of the third step, the codes and accompanying data extracts were analyzed and integrated by AP and HC to form preliminary secondary codes and then main themes. Where appropriate, text segments were assigned to multiple codes and themes. To ensure rigor, themes and sub-themes were reviewed by the second author during the fourth step. This involved revisiting the raw data and discussing the analysis with to determine whether certain themes should be merged or divided. The penultimate step concerned AP and HC verifying and finalizing the themes and naming them appropriately to ensure that they accurately reflected the raw data and codes. In the final step, AP and HC discussed the themes, sub-themes, and underlying codes insofar as how they provided insight into lived experience of serving members of the UK armed forces coping with gambling problems, alcohol use, and mental health help-seeking difficulties. In the event of differing opinions, AP and HC discussed their personal insights of the research findings in a supervisory meeting; consideration was given to whether there were any beliefs or experiences that may have predisposed the interpretation of the data toward a particular conclusion. Additionally, SD was provided with exemplars of verbatim quotations from the data and reviewed AP and HC's interpretations to ensure that these patterns were evidenced in the raw data. These processes were followed by a team discussion to ensure a consensus had been reached. Specific data extracts were also selected as quotations for the final manuscript writing (23).

## Results

Four major themes were identified: (1) Harmful and protective occupational factors, (2) Socio-cultural and personal influences on harmful behaviors, (3) Organizational attitudes toward mental health and help-seeking, and (4) Current support pathways and provision. These themes, accompanying subthemes and supporting verbatim quotes as they related to gambling and wellbeing are presented in Table 2.

**Table 2:** Description of themes.

**Theme**	**Sample Quotes**
1. **Harmful and protective occupational factors** *Sub-themes:* •Service-related stressors •Factors promoting wellbeing	It started from overwork…I was working from 7 in the morning till 10 at night for months. I was at breaking point and I started having panic attacks, I become withdrawn, I used to shake all the time and then thoughts of suicide came in until I attempted to take my own life (Participant 5) You've got access to cheap and readily accessible alcohol. If you are a gambler, the living costs aren't very much your food costs isn't very much - that means you've got more money to spend on gambling. Before you know it becomes noticeably a problem (Participant 6)
2. **Socio-cultural and personal influences on harmful behaviors** Sub-themes: •Organizational social norms and a culture of acceptance •Internal, generational lifestyle changes	In the job I had before I joined, you go to work and then you go home. Whereas because the RAF is very sociable; it's one of those things if someone in your kind of group, especially if you look up to him as well and they're doing certain things [gambling], as a young buck you want to be in that group (Participant 6) If you don't drink in the bar, then you don't make the connections necessarily that other people might. No, you don't need to drink to succeed, but a lot of our socialization and a lot of work gets done in the bar, you know, over a pint… it's a pivotal part of our lifestyle (Participant 9)
3. **Organizational attitudes toward mental health and help-seeking** Sub-themes: •A more open culture •Hierarchical stigma and harmful attitudes •Lack of recognition and shame	The frontline bosses, they are an older generation where they've been brought up as “pull your socks up, you know you're a man, you can't cry” - you know that kind of generation (Participant 17) If I say, “hang on a minute I'm struggling a bit here” I am just wary they might say “well don't give him too much work or don't let him do that intense project because he's struggling at the moment, we don't want to put him under more work pressure” (Participant 6)
4. **Current support pathways and provision** Sub-themes: •Barriers to getting the right support •Lack of action and impact Effective support systems	It makes you feel really bad inside and a bit guilty about losing so much money… It makes you feel depressed, and I've come home before and harmed myself before because of my gambling (Participant 7) There are several strands of support that you can access but I've seen some people just get fobbed off…Or get signposted to go and speak to someone but actually that's the wrong person…Or they'll get passed onto this person. That's happened to me a few times where I've gone for some help from the med center and they've referred me on to DCMH, but the person who I got put in contact with at DCMH didn't have much of an understanding of my issue (Participant 7)

### Theme 1: Harmful and protective occupational factors

#### Coping with service-related stressors

Stress caused by a high workloads and long shifts, often intensified by a sense of responsibility and expectation to perform well, was widely discussed. Stress contributed to various psychological problems including burnout, anxiety, suicidal thoughts and behaviors, panic attacks, and the inability to undertake everyday tasks. Increased gambling and alcohol intake were also used as stress-coping.

“*I was definitely suffering with some anxiety issues…Our* [trade] *is … quite stressful because a lot of the decisions you're making have got safety implications…For a period of time my resilience had been depleted”* (P1).

Separation from family was identified as another significant occupational stressor. From an individual viewpoint, it caused loneliness and a deterioration in existing mental health issues. More broadly, being absent for long periods during deployments and the disruptive nature of frequently moving around put a strain on relationships with spouses, partners, and children. These difficulties were sometimes intensified by not being able to communicate with loved ones while overseas and the inability of some UK bases to provide adequate accommodation for visiting families.

“*Location can be difficult…I think probably one of the things that contributed to speeding up the end of my last relationship was the fact that I lived 2 h away. I was away Monday to Friday and home at the weekends”* (P1).

In some cases, traumatic experiences in Afghanistan and Iraq manifested into PTSD symptomology and psychological distress, resulting in heavy alcohol consumption to “*self-medicate*” (P6). Moreover, it was reasoned that “*walking around with rifles and among landmines*” (P7) during deployments meant that activities such as gambling were considered comparatively very low risk, leading to increased participation in gambling by several participants.

“*It was when I came back, I saw the issues… I got back into the normal environment, I was going out a lot, drinking a lot. I tended I got into this little… thing where I would like literally go home and then just sit at the table and continue drinking, then cry uncontrollably”* (P6).

The tedium of being deployed and living on remote bases where “*there is nothing to do*” (P17) was linked to increased alcohol consumption and gambling activities. Likewise, elevated betting and drinking due to boredom were reported among trades with significant periods of free time (such as fire and rescue service roles), and individuals unable to work due to injury. These behaviors were further reinforced by having easy access to cheap alcohol, online betting apps, and a high income with low expenditure.

“*There was a base that was quite well renowned for a big alcohol culture because everyone was just stuck there and it would be ‘well, the only thing to do is finish work at 5 o'clock and go straight to the bar'. So, I think that's probably the location element…. There isn't a great deal to do down the Falklands”* (P12).

“*It's* [gambling] *easily accessible nowadays with smartphones and smart devices, so you can always play on a whim … with being away it's based on the boredom and it's almost an instant high which replaces some of the feeling of loneliness”* (P10).

“*You've got access to cheap and readily accessible alcohol. If you are a gambler, well the living costs aren't very much, your food costs isn't very much - that means you've got more money to spend on gambling. Before you know it becomes noticeably a problem”* (P8).

#### Coping with COVID-19-related stressors

Elevated levels of anxiety and low mood due to COVID-19 restrictions affected most participants which was often exacerbated by RAF environments. One of the biggest difficulties related to residing on RAF bases and being confined to a single room so small its inhabitant could “*touch everything from where I am sat*” (P11). Wellbeing was further impacted by the perceived lack of clear top-down communication and the use of the “*military discipline system unnecessarily and unfairly*” (P3) at certain bases in response to the pandemic, while offering little support to personnel.

“*There's been a steady decline* [mentally] *during the lockdowns… I would have thought that we would have had a better understanding of how to control an infection amongst personnel in stations rather than just saying “right you are all locked in your room and then we'll leave you there for weeks on end”* (P8).

Moreover, increased gambling and alcohol consumption had affected more than half of the participants during lockdowns due to boredom of being forced to work from home or to remain in their rooms on base and/or having to undertake more desk-based tasks rather than practical, high-risk assignments. Having even more disposable income than usual due to travel and social restrictions further enabled these behaviors.

“*I've noticed I started playing scratchcards a lot more often. You're not frightened to download that extra 10 or maybe an extra 20 quid because I no longer have to travel back home and pay the fuel, and all that sort of thing, so expenditure per month has decreased”* (P11).

“*I have gambled a little bit more since Covid…daily sport accumulators… it's when you're at home, or when I've been in isolation; its boredom”* (P5).

#### Occupational factors promoting wellbeing

Numerous benefits and protective factors of the job were revealed by participants. They felt that RAF had equipped them to be confident, resilient, determined, and flexible individuals; skills which could be transferred to other areas of their lives. For some, deployments provided career and personal opportunities, such as visiting new places, learning new skills, and having a “*break from reality*” (P5). Serving within a network of “*like-minded*” (P3), supportive people was also invaluable to personnel's wellbeing.

“*The experiences I've had and the confidence and the development opportunities it's given me far outweigh any kind of negatives…I think the support network is really good…I think the relationships and the friendships you build in the military are much tighter in a shorter period of time than they would be in the civvy world”* (P1).

Other benefits were more salient than serving with “like-minded” supportive people – mainly because of the collective range of “other benefits” mentioned (e.g., good accommodation, confidence, grit, resilience, determination, important skills, deployments and the opportunity to travel, a change of scene, chance to have a break from everyday life, good pay, stable employment, instant access to healthcare, early intervention, access to other amenities such as the gym, sports facilities).

The RAF was also described as offering stable employment, excellent healthcare, and good access to sports and educational amenities. Indeed, it was described as a “*positive lifestyle*” (P5) which other employers do not or cannot offer.

### Theme 2: Socio-cultural and personal influences on harmful behaviors

#### Organizational social norms, gambling, alcohol, and a culture of acceptance

Traditionally, many social RAF events revolved around alcohol consumption and gambling. With regards to the former, “beer calls”, “initiations”, and informal social gatherings at RAF base pubs had become a significant part of military culture. In the context of betting, the RAF sports lottery, and days out to the horse races were commonplace. As such, these activities were widely accepted and expected.

“*I think the military are big on tradition and culture. Rather than seeing it as a problem it's ‘oh it's always been a drinking thing that's what we always do”* (P16).

“*There is kind of a casual attitude to gambling…There are some slot machines on bases that are higher value…they aren't the couple of hundred pound pay out ones, they are the hundred pound or pay-out ones. I always remember being on them at tea break”* (P7).

A main motivation for engaging in these activities was not wanted to miss out on key social events, which were valued facilitators in fostering the camaraderie and a sense of belonging so integral to the RAF. One participant asserted that it could be isolating and lonely abstaining from alcohol in the air force. General discussion between colleagues about bets, accumulators, and tip sharing were also thought to be particularly common as an easy conversation starter and way of socializing.

“*I like to go to the races, have a few drinks and have a few bets…It's a bit of a culture thing…a lot of the guys at work love the races and having the bets. You're watching football together in a group and you're having a few drinks and you're betting on the next goal scorer...”* (P14).

The desire for social acceptance and increased exposure to potentially harmful behaviors while serving in the RAF had heavily influenced many participants' gambling behavior and alcohol use. This was especially the case in their early years of service when they were keen to mirror the behavior of their peers and superiors. Frequent heavy drinking sessions often ensued, with accounts of many personnel, including participants themselves, being hungover on duty. Others witnessed colleagues lose considerable amounts of money through bets they put on with their colleagues while drinking. Importantly, these behaviors were seldom perceived as particularly problematic or risky.

“*There was a year a year or two, where I did start drinking a lot more… You know, the friendships going on at that time and drinking naturally did become a little bit of an issue… I found myself drinking more often on a weeknight and waking up for work the next day unfit for work. I'd do that several times a week”* (P11).

“*In the job I had before I joined, you go to work and then you go home. Whereas because the RAF is very sociable, it's one of those things if someone in your kind of group, especially if you look up to him as well and they're doing certain things like gambling, as a young buck you want to be in that group, so you think you want to kind of socialize with them in that way as well”* (P6).

Yet, some were of the view that although a culture of alcohol consumption and gambling continued to exist within the RAF, it did so to a much lesser extent nowadays. Changes to RAF rules and guidance were pinpointed as contributing to reversing traditional social norms and included: expectations to have a more professional attitude; discouraging the use of fruit/slot machines including, according to some participants, their removal from bases (despite this is not being current policy); alcohol limits/bans while on deployment; and disciplinary action for bad behavior relating to alcohol consumption.

Despite this, long-term impacts of the RAF gambling culture continued to affect several participants. They explained that using the slot machines on RAF bases started as a largely social, affordable habit which become a normal part of their life. Once the use of slot machines was more actively discouraged and became a less popular social activity at their bases, they found it difficult to simply stop gambling and instead began using online apps or going to bookmakers alone. Engaging in these activities in isolation saw an increased focus on the “*buzz*” of winning and made it easier to “*get lost in the gambling world*” (P7) and hide it from others – all of which contributed to the gambling becoming more harmful.

“*I joined when I was 17 and a half … there were some slot machines on bases… I'd play on them on tea break with friends … then I remember going into bookmakers just to see because I had never really been in a bookies before… I saw loads of slots so I put some money in and I won 500 pounds… And then I remember going back in and playing again…I then had some money in this online betting app… At times it has been out of my control…I'm definitely after a win, to outsmart this machine”* (P7).

#### External, generational and lifestyle changes, gambling, and alcohol

External factors were identified as driving perceived cultural changes around harmful behaviors. Younger RAF personnel were thought to be less likely to engage in heavy drinking sessions with their peers. Instead, they had a “*healthier attitude*” (P1) toward alcohol and took greater interest in going to the gym and playing video games.

“*I think it's a different kind of generation, “PlayStation generation” as I call them. They like to be indoor gaming and not getting up to mischief”* (P17).

Conversely, it was speculated that substance use was more widespread among junior officers due to social norms and the popularity of attending the gym. Indeed, it was reasoned that the pressure younger personnel feel to attain a physical ideal may have instigated an increased use of steroids and other performance enhancing drugs.

“*With the culture, everything you see online is about fitness these days rather than anything else and trying to keep up an image and pressure in the military where you are meant to be physically fit, I can see why folk would turn to supplements”* (P16).

The rise in popularity of online betting apps was also thought to have encouraged gambling among younger personnel, who spent large amounts of time on their smartphones and were being targeted by betting companies in this context. Engagement with these platforms were also said to have increased more generally during the pandemic in lieu of going to the bookmakers, which some participants had continued to use as restrictions lifted.

“*I've noticed in the last couple of years…Paddy Power, Bet Fair, of the big gambling groups that have an app and target young men through advertising… This is part of we are reflection society - we're not excluded from it*” (P8).

Changes in participants' personal lives, such as getting married, having children, and buying their own property had initiated a conscious effort to modify their behaviors. Specifically, they had reduced or stopped gambling or drinking alcohol in response to their new priorities, thereby prompting a wider cultural shift within the RAF. Furthermore, the growing number of personnel now living off base meant it was more socially acceptable to have non-alcoholic beverages during work social events to drive home safely. Others had cut back on their alcohol consumption simply due to growing tired of making “*a fool*” (P7) of themselves and being hungover in work.

“*The social interaction and turning up to the bar for someone's leaving do or arrivals drinks is still very much there, but…it's perfectly accepted that you're going to have two pints of coke because you're driving home…The focus has switched to even if you're not going to have a drink, come along and you can still have good interactions”* (P12).

“*In a past life, I was single, I did have a lot of spare money so I wouldn't be bothered if I lost a bet. But now I'm a bit more conscious…my wife will say “oh shall we buy some new garden furniture?”* (P6).

### Theme 3: Organizational attitudes toward mental health and help-seeking

#### A more open organizational culture

There was a noticeable effort by the RAF to acknowledge and promote the mental health of its personnel over the past 5–10 years. The provision of RAF-run training and educational programmes were identified as key facilitators, which included mental health first aid courses, trauma risk management, self-esteem classes, and gambling awareness sessions (GamCare). The RAF was also praised for working closely with charities such as The RAF Benevolent Fund (RAFBF) and the Soldiers, Sailors, Airmen and Families Association (SSAFA), as well as undertaking staff mental health surveys.

“*I think it* [mental health] *is very focused on. I've actually done the mental health first aiders course; they are always providing some sort of help and assistance in there, and these courses are always available for people to jump on”* (P11).

“*I know that the air force work a lot with something called GamCare for awareness”* (P9).

These initiatives were thought to have fostered a greater organizational understanding that “*mental health is all encompassing and can affect many, many areas of someone's life*” (P4). Consequently, there were increased efforts on the ground to “*make a point of checking in on*” (P10) each other informally and normalizing conversations about mental health – both of which often encouraged formal support-seeking.

“*I've been in nearly 22 years now, and it has changed a lot. It used to be that if you got any mental health issues in the air force you were or cast aside whereas now everybody knows mental health is such a wide range of issues. Everybody's accepting it and getting more and more approachable with it”* (P4).

#### Hierarchical stigma and harmful attitudes

The RAF was described as having “*a long way to go*” (P7) to reduce organizational stigma around mental health and occupational barriers to help-seeking. Personnel were expected to formally communicate wellbeing issues to their manager initially. However, not everyone felt comfortable doing so because their superiors were not considered “*approachable*” nor “*compassionate*” (P15). Others described their manager as being too busy or unaware to initiate these conversations. This protocol also made it difficult to intervene or raise concern about another colleague of the same or higher rank. Worryingly, several participants felt minimized and belittled when they had disclosed their mental health struggles.

“*I had one tour away that was more difficult… I was very unsupported… it is not very often that your manager just pulls you in themselves and has a conversation with you just to make sure you're okay”* (P16).

The military identity – conceptualized by stoicism, putting a “*brave face on*” and having a “*gung-ho attitude*” (P4) - additionally led to the concealment of problems. The stereotype – which affected personnel regardless of their gender - caused concern about being perceived as weak if they admitted to having mental health struggles.

“*Stigma and fear will stop people reaching out… I think it's probably a military thing …the ‘stiff upper lip' and ‘soak it up and man up' is what you'll hear a lot… I think the natural culture that it brings probably doesn't help because they don't want to be seen as weak in front of the hierarchy's eyes or in terms of their peers”* (P9).

A perceived absence of independent support services provided *via* the RAF and the subsequent lack of separation between help-seeking and personnel's employment also caused trepidation. Specifically, it was feared that any disclosures would be detrimental to future career progression, promotions, passing vetting procedures, and/or the type of work assigned, which was said to already be challenging “*in a very hierarchical organization*” (P8).

“*One of the awkward things with the med process in the air force is that it is tied in so closely to your job, and rightly so, but the problem is every time you go to the Med Center you know full well that's going to go into your medical records. I suppose there is that concern that further down the line it's going to come and bite you”* (P1).

#### Lack of recognition and shame

Many mental-health related issues reportedly remained overlooked because the RAF is “*very good at pushing things under the carpet*” and has a culture of “*if we don't talk about it is not there*” (P16). This was thought to have impacted general understanding about these topics, distorted organizational prevalence rates, and prevented access to early intervention. This was frequently discussed in the context of gambling insofar as such problems within the RAF were not initially thought to be particularly high. However, some participants later reasoned it was easy to hide because it was not recognized, talked about, and addressed as much as it should be.

“*From work from my experience, I'd say gambling* [in the RAF] *is a niche issue…But it might be a fundamental lack of appreciation for the actual problem, and it might affect more people than I ever dreamt possible”* (P9).

This lack of recognition and understanding left some participants feeling too embarrassed to admit their issues. This was the case across the mental health spectrum, but shame was particularly common among those who had experienced problem and moderate-risk gambling. The prospect of admitting to considerable financial loses and control over their actions was described as especially humiliating, as was concern they would be perceived as “*stupid*” (P13) and unable to do their job.

“*Even now there's only my wife that knows about my gambling… I know that there is support available, but I think for a lot of people like myself… I wouldn't want the RAF to know. I wouldn't want lots of people to know, because it's not something I'm proud of… I was embarrassed more than anything”* (P13).

“*I reckon the Air Force would look at you and think ‘well you can't manage your money or yourself, how can you manage a section?”* (P5).

As pertains to other addictive behaviors, the “*taboo”* and “*hidden”* (P9) nature of illicit drug use was attributed to the RAF's zero-tolerance policy. Namely, the RAF was criticized for being “*more likely to kick someone out*” for drug use rather than “*trying to fix the underlying problem*” (P5), thereby causing individuals to feel too scared and ashamed to ask for help.

“*There is a massive drug culture in the military…You may have done it once or twice around a group people and then you'd maybe struggle to stop doing it. And because it's zero tolerance, people know that they'll get kicked out if they do talk about it which makes it hard to…ask for help”* (P16).

Overall, participants felt that a similar sense of stigma and shame surrounded gambling harm and acted as a barrier to help-seeking.

### Theme 4: Current support pathways and provision

#### Barriers to getting the right gambling and wellbeing support

There was general awareness of where to access help for mental health and wellbeing-related problems within the RAF. The GP, Padre service, and charities such as RAFA (The Royal Air Forces Association), SSAFA, and the RAFBF were considered first contacts who would signpost or refer where necessary. Furthermore, there was familiarity with the Department of Community Mental Health (DCMH), which several participants had personally accessed.

However, there was a sense of not “*knowing where to start*” (P16), along with a “*lack of connectivity*” (P7) between generic and specialist services. Consequently, the process of securing support could be overwhelming and confusing. There were accounts of being given the wrong contact details, being “*passed from person to person*” (P7) and feeling overwhelmed about selecting which service(s) to access. It was suggested that personnel needed proactive assistance with making this first step.

“*I think with the number of options available, perhaps a central hub where we can put in questions and it sort of links up to all these elements, you know, rather than phoning the SSAFA and them saying ‘well that's not quite us.' Maybe like a triage system…”* (P11).

Very few could identify specific support provision for problems with harmful behaviors, either voluntarily or when explicitly asked. Furthermore, those who were aware of gambling-related help criticized it for merely focusing on financial management. This was problematic because most participants with lived experience did not encounter financial issues due to their salaries and low expenses. Instead, psychosocial impacts such as guilt, depression, self-harm, and relationship problems were prevalent –although reportedly seldom understood among informal and formal support networks.

“*It makes you feel really bad inside and a bit guilty about losing so much money… It makes you feel depressed, and I've come home before and harmed myself before because of my gambling - that's one thing that I have done on several occasions because I have lost more than I wanted to”* (P7).

“*I wouldn't know where to* [signpost someone for gambling-related issues]. *I'm in a peer support job… if people were to come to you with a gambling problem you wouldn't necessarily link the two… you just wouldn't associate it with a mental health issue…I'm not sure if any of our policies would lead you down the lines of checking why* [the issues occurred*], most of ours are fixing the problem rather than why the problem occurred”* (P10).

“*There's still a bit of a stigma in my honest opinion that if someone did say ‘I've got a gambling problem', people wouldn't go ‘oh you got mental health illness.' They would think about more the monetary side first rather than the person”* (P6).

Another reported issue was that mental health practitioners did not always have lived experience which hindered their ability to relate to and truly understand their service user's situation. Similarly, a desire for more practitioners to have first-hand knowledge of the “*idiosyncrasies*” (P12) of working for the air force was expressed. Therefore, several participants felt increased peer-based mental health services would enable better quality and individualized support.

#### Lack of action and impact

Despite the RAF “*saying the right things*” (P9) around mental health, it was argued that this was not necessarily being translated into tangible change and effective support on the ground. Instead, current approaches were described as “*a tick box*” (P9) exercise without “*any passion*” (P15) to genuinely help people. Perceived lack of proactivity leading to missed opportunities was a particular issue raised by interviewees, such as the failure to explore mental health and issues relating harmful behaviors as part of annual medicals. Lack of action around addressing issues with the chain of command's stigmatizing attitudes was also a source of frustration, thus a greater provision of in-depth mental health and wellbeing management training was highlighted.

“*I would put workshops into the promotion courses…Not just an hour session…A day where you talk about things like* [mental health]. *It's all about looking after your lads and lasses and it's an awareness thing, and an understanding that's key”* (P5).

#### Effective gambling and wellbeing support services

Many of the participants who had accessed support *via* the RAF described a range of positive experiences. Despite the number of issues raised around the chain of command, there were also examples of the invaluable support offered *via* managers and warrant officers. These individuals tended to be personable, understanding, and empathetic. They were also likely to take appropriate proactive action, such as making referrals and attempting to reduce work-related stressors. However, it was reiterated that the extent of a manager's supportiveness was “*person-dependent*” (P15).

“*I've been quite lucky…I have good management around me; they were always really supportive and listened to you and actually checked in to see how you were… I think a big part of this comes down to who your managers are if you've got good people that you work with”* (P16).

Many who had received formal mental health support (namely, *via* the DCMH) felt more able to cope, less alone, and noticed an overall improvement in their wellbeing. Common components such as feeling listened to, having ample time and effort put into them, and feeling safe in a confidential setting were also integral to these positive outcomes.

“*I went through the DCMH process and had some really good Cognitive Behavior Therapy with them at* [base name], *which was really useful. I think for me doing that was just a really great way to sort of reset… You think you're the only person in the world that's suffering but when you actually understand that quite a lot of people seem to feel the same way…I found that personally quite helpful”* (P1).

Around half of participants disclosed that personal issues had impacted their mental health and were grateful for the support they received. Others were able to obtain assistance with practical issues associated with their job such as changing their job within the RAF, rescheduling planned deployments and help with financial issues. The welfare support provided during deployment for both personnel and their families was additionally commended by several participants.

“*I had issues with concentration and sleep for a period …I probably would more likely drink more when I was going through my divorce… I got lots of support from where I was working, and they even managed to get me moved closer to where they* [their children] *were living”* (P2).

“*Chasing losses* [from gambling] *was just oppressing, just pushing me down and thinking I can't get out of this… But The Benevolent Fund were actually fantastic… I moved into SFA housing and they gave me £500 to buy some white goods”* (P14).

Interviews revealed that informal social support from fellow personnel should not be underestimated. Indeed, many participants continually found solace when confiding in their peers, who they felt could relate to their experiences.

“*I just talk to people…you know military friends who can relate to it* [PTSD]…*it's chatting to people who can relate to it…if you chat to people who have suffered its quite therapeutic”* (P5).

## Discussion

The present study explored the lived experience of mental health help-seeking, gambling problems, and alcohol use among members of the RAF. Four key themes were identified, which together portrayed occupational influences on mental health, harmful behaviors, and help-seeking within the RAF. Work-related stressors, traumatic experiences, traditional vs. modern organizational cultures, stigma, resilience, and support provision were among the key features discussed by participants in this context. To our knowledge, this is the first such qualitative investigation of these topics with active-duty military personnel. Our findings indicated that alcohol consumption and problem gambling were prevalent within the RAF, as were mental health problems related to stress, anxiety, depression, and PTSD. This is consistent with research identifying elevated vulnerability to these problems among current and ex-military personnel compared with the general population (1, 12, 24–26). Let us now consider the four main themes identified.

The first theme revealed that participants struggled with various aspects of military life, which increased their susceptibility to co-occurring poor mental health and increased engagement in harmful behaviors (27). Indeed, alcohol consumption and gambling were sometimes used as maladaptive coping responses to high workloads, deployments, trauma during combat, frequent relocations, boredom during quiet periods and injury, and living on bases during COVID-19 lockdowns. These stressors often led to feelings of isolation and hopelessness, while the impacts of working away conflicted with family life and relationships. This also indicates that the perceived extremes of RAF life - dangerous work with long hours and periods of boredom - could be equally as damaging and manifest similar maladaptive coping mechanisms.

Previous research has found associations between traumatic events during international military operations, mental health issues, alcohol (28) and gambling problems (25) among military veterans. Generally, however, associations between mental health problems (especially PTSD) arising from military experience and the impact on gambling behavior remains under examined (12, 25). On the other hand, the protective nature of service life has been shown to be associated with reduced vulnerability to mental health problems (29, 30) and this was evident in the participants' experiences. Specifically, the RAF was praised for equipping personnel with a range of key skills, offering excellent employment privileges, fostering camaraderie, and instilling a sense of pride in serving for the air force. Together, these factors enabled personnel to cope with adversity, both in their professional and private lives. This is an important finding as it highlights that protective influences from serving with the RAF can be extremely impactful. Moreover, it emphasizes the importance of continuing to provide and improve organizational training programmes which promote psychological resilience, especially in response to stress and trauma (31).

The second theme highlighted the role of cultural normalization and peer influence on alcohol consumption and gambling within the RAF. Participants felt that conforming to social pressures to build camaraderie and impress the hierarchy drove and maintained these social norms. Previous studies indicate these factors have been instrumental in normalizing group bonding over alcohol (32, 33). Participants' accounts of increased engagement in these activities as a direct result of joining the military insofar as drinking, gambling on slot machines and making bets with colleagues (and more recently *via* online gambling apps) highlighted the paradox of the legitimatisation of harmful behaviors and the military identity of displaying discipline, physical health, and mental toughness.

Importantly, for some, what began as relatively harmless drinking and gambling sessions to facilitate a sense of belonging sometimes became maladaptive, affecting their physical and mental health (34). Moreover, the observation that problematic behaviors often went unrecognized by individuals themselves, their peers, and chain of command indicates how the normalization of such activities has negatively impacted the likelihood of detection prior to becoming a potential crisis (32). Understanding and evaluating the emotional, physical, and economic long-term implications of delayed identification of problems relating to alcohol and gambling among serving military personnel would be beneficial to explore in future studies.

Our findings do suggest however that the culture and perception toward alcohol and gambling in the RAF is shifting. This was thought to have been driven by organizational influences - namely the RAF no longer promotes or actively dissuades these activities - and personal lifestyle factors, such as the desire to be more responsible and healthier for themselves and/or their family. In contrast, there was concern that problems among younger officers living on base were not being detected by others because they were less likely to socialize outside of their rooms and may instead engage in harmful behaviors in private. Although these observations were mainly anecdotal, the cross-sectional study from which this qualitative element is derived, found that gambling problem risk was associated with younger age (4). Moreover, recent findings show that significant correlates of increased gambling during the COVID-19 pandemic were found among those younger aged male members of the general population with increased problem gambling severity scores (35, 36).

Although Theme 3 highlighted improvements in the availability of mental health education and training within the RAF, notable barriers to help-seeking remained. Specifically, participants raised concerns about perceptions of mental health stigma and fears that disclosure of difficulties or seeking mental health services may negatively influence one's military career. These reasons have been identified elsewhere within UK military samples (37).

Different types of stigma were reflected in participant accounts. In the context of the RAF, public stigma related to concerns of being perceived as less competent, belittled, or dismissed by more senior ranks and colleagues. Internalized stigma related to individuals with mental health difficulties, or gambling problems internalizing the negative stereotypes held by others, which evoked strong feelings of shame, and perceiving themselves as “weak” (18). These stigmatizing beliefs were coupled with concerns about the potential damaging impact that admitting a mental health difficulty or gambling problem could have on their career; reflecting structural stigma, where individuals are (un)intentionally disadvantaged due to having mental health difficulties or gambling problems (38).

Despite efforts to create an open culture to discuss wellbeing issues, considerable stigma and barriers continued to exist for those experiencing mental health and gambling difficulties in the RAF. Importantly, however, while several participants did eventually access support for their mental health needs, none reported seeking help for their gambling problems. Future research needs to explore help-seeking across a broader range of wellbeing, as the literature is predominately focused upon mental health, with less attention given to help-seeking for gambling or substance use.

Theme four highlighted the experiences of personnel in identifying and accessing the current support provisions available for their wellbeing. Personnel identified practical barriers centered around a lack of knowledge of the pathways available to access specific support (e.g., gambling support). Practical barriers to treatment have been noted elsewhere among ex-military personnel (17). Participants also raised concerns and criticized provision for failing to consider the idiosyncrasies of working for the RAF, which meant support was not tailored to account for the unique context of military life. Personnel preferred the RAF to adopt a proactive approach, such as using annual medicals and pre- and post-deployment reviews as opportunities to conduct screening, ask about wellbeing, and signpost appropriately. Despite these challenges, several participants described the formal mental health support they had received positively and acknowledged these experiences had increased the likelihood they would access future services earlier than they had done previously. Further research is needed to better understand barriers and obstacles to help-seeking in the military.

## Limitations

It is important to note that interviews were conducted during the COVID-19 pandemic in 2021 when the UK was subject to public health protection measures which may have influenced individuals' responses. As the global impact of the pandemic eases, future research should seek to gain a fuller understanding of how COVID-19 continues to affect the lived experience of gambling among military personnel. The generalisability of findings may be limited as the personnel were self-selected. Additionally, because some participants had accessed support for their mental health, their experiences of wellbeing in the RAF may differ from those who had not sought help. All participants were of white ethnicity and thus the experiences of people of color were not captured. Notwithstanding these limitations, the present study did reflect the lived experience of individuals from across a range of gambling experience (PGSI scores) and genders.

## Conclusions and future research

The present qualitative investigation identified work-related stressors, traumatic experiences, traditional vs. modern organizational cultures, stigma, resilience, and support provision as key features of the lived experience of serving RAF personnel. Our findings indicated that alcohol consumption and gambling problems were commonplace within the RAF, as were mental health problems related to stress, anxiety, depression, and PTSD. Taken together, this study highlights the interplay between vulnerability to harmful gambling and mental health coping among serving military personnel.

Future research should continue to evaluate how best to screen and support personnel with co-existing gambling problems and mental health challenges. It is notable that at present no such screening or assessment procedure exists within the UK Armed Forces community, despite an established evidence base on the best diagnostic tools that could be used [e.g., (39)]. By contrast, the US military has undertaken annual screening since 2018 of active service personnel and routinely screens for gambling problems prior to deployment. Improving the accessibility of safer gambling information within the Armed Forces, screening for potential harm, and developing specialist treatment and support for service personnel and affected others should be prioritized.

Finally, a great deal more research is needed to identify and overcome the military-specific barriers to help-seeking. Imparting information to normalize and seeking help, such as through the role of peer mentoring, needs to be facilitated within the Armed Forces community. This could be done by, for instance, normalization through personal stories of others who had difficulties and then sought help, written stories, speakers at in-person events, and using statistics to demonstrate the prevalence of various concerns and help-seeking behaviors. This would harness the social connection that is so vital within the RAF and would enable people to connect with someone who they often perceive as ‘like-minded' but also enables them to see that if they have struggled and worked through it by seeking support, then this is also an option for them. Doing so would harness the strong social connections that exists within the RAF and may facilitate culture-change through the lived experience of individuals comfortable to share their stories for others to hear.

## Data availability statement

The datasets presented in this article are not readily available because due to the nature of this research, participants of this study did not agree for their data to be shared publicly, so supporting data is not available. Requests to access the datasets should be directed to s.o.dymond@swansea.ac.uk.

## Ethics statement

The studies involving human participants were reviewed and approved by the Ministry of Defence Research Ethics Committee (Reference: 1051/MoDREC/20). The patients/participants provided their written informed consent to participate in this study.

## Author contributions

HC, AP, GD, and SD contributed to conception and design of the study. HC transcribed the interviews and performed thematic analysis. AP transcribed the interviews and HC with the support of AP performed thematic analysis. All authors wrote sections of the manuscript, contributed to revisions, and approved the submitted version.
