# Supplementary material for: Gambling problems and help-seeking in serving United Kingdom military personnel: A qualitative study
Source: Front Psychiatry. 2022 Dec 23;13:1003457. doi: 10.3389/fpsyt.2022.1003457 (PMC9816802; doi:10.3389/fpsyt.2022.1003457)
Supplement: Supplementary file 1 [file Data_Sheet_1.docx]

**Gambling problems in serving United Kingdom military personnel:**

**A qualitative study**

Hannah Champion, Amy Pritchard, Glen Dighton, & Simon Dymond

**TOPIC GUIDE AND INTERVIEW QUESTIONS**

**Interview Schedule**

Okay, now to get started, we want to discuss some of the measures you completed within the survey in some more detail. We are particularly interested in how COVID has impacted the wellbeing and coping of RAF personnel so we

**COVID Questions**

1. Has COVID-19 influenced your mental health? If so, how?
2. Has COVID-19 influenced your gambling behaviours? If so, how? (Prompts - lockdown, mental health, change in financial status)
3. Do you think your alcohol use has been impacted by COVID? If so, how?

**So now moving on to focus on your mental health specifically.**

**Mental Health Experience**

1. Did you have any difficulties with your mental health prior to COVID?
2. If so, when did these difficulties start and can you describe the difficulties you experienced?
3. Did these occur prior to you joining the RAF, or during your enlistment?

*The context of the RAF*

1. Has joining the RAF affected your mental health in any way? If so, how?
2. Are there any specific experiences of being a serving RAF member that have influenced your mental health?
3. Thinking back over your years of service, are there any features unique to serving in the RAF which may have impacted on how you coped with your mental health? If so, what might they be?
4. What is the culture surrounding mental health within the serving RAF community?
5. Has your mental health ever influenced your ability to do your job within the RAF? If so, how?

*Treatment/support*

1. Tell me about what sources of support you think are available to support RAF personnel with their mental health?
2. Do you think there is a need for mental health services/support within the RAF cohort?
3. Suppose you were in charge, what more do you think needs to be done regarding mental health within the RAF?

**So now moving on to consider your gambling behaviour.**

**Gambling Experience**

1. When did you first start gambling?
2. What type of gambling was it?
3. Was there was a reason you started gambling?
4. What is the nature and extent of your experience with gambling more recently?
5. Have you personally experienced any difficulties/challenges that are associated with gambling? If so, what are these?
6. Has anyone else experience any difficulties/challenges associated with your gambling? If so, what are these?

*The Context of the RAF*

1. Did joining the RAF influence your gambling behaviour?
2. Was there a particular experience in the RAF that influenced your gambling behaviour, or caused you to start gambling?
3. Thinking back over your years of service, are there any features unique to serving in the RAF which may have impacted on how you coped with gambling? If so, what might they be?
4. What is the culture/attitude towards gambling within the RAF community?
5. Has your gambling ever influenced your ability to do your job within the RAF? If so, how?

*Treatment and support*

1. Tell me about what sources of support you think are available for gambling related difficulties?
2. Do you think there is a need for gambling related services/support within the RAF cohort?
3. Would you access services for support with your gambling?
4. Suppose you were in charge, what more do you think needs to be done?

**So now moving on to consider your alcohol and substance use.**

**Alcohol/Drug Experience**

1. Do you drink alcohol? / Do you use illicit drugs?
2. Has your alcohol or drug use ever posed any problems for you?
3. Did you drink alcohol or use illicit drugs prior to joining the RAF?

*The Context of the RAF*

1. Thinking back over your years of service, are there any features unique to serving in the RAF which may have impacted how you use alcohol or drugs? If so, what might they be?
2. What is the culture/attitude towards alcohol and drug use within the RAF community?
3. Has your drinking or drug use ever influenced your ability to do your job within the RAF? If so, how?

*Treatment and support*

1. Tell me about what sources of support you think are available for alcohol or drug related difficulties?
2. Do you think there is a need for substance services/support within the RAF cohort?
3. Suppose you were in charge, what more do you think needs to be done?

**Closing section**

We are almost out of time and will have to end the interview soon. Again, let me thank you on behalf of the research team for sharing your experience with us here today.

As we outlined in the consent form, today’s session was recorded, and we will transcribe all content before conducting what is called a thematic analysis on the contents of discussion to identify overlapping themes in your experience with other interview participants.

Would you like to discuss any further points or add anything further?

We have a shopping voucher for you, as a thank you for your time.
